# Genome-wide association study of psychiatric and substance use comorbidity in Mexican individuals

**DOI:** 10.1038/s41598-021-85881-4

**Published:** 2021-03-24

**Authors:** José Jaime Martínez-Magaña, Alma Delia Genis-Mendoza, Jorge Ameth Villatoro Velázquez, Marycarmen Bustos-Gamiño, Isela Esther Juárez-Rojop, Carlos Alfonso Tovilla-Zarate, Emmanuel Sarmiento, Erasmo Saucedo, Oscar Rodríguez-Mayoral, Clara Fleiz-Bautista, Beatriz Camarena, Alejandro Aguilar, Thelma Beatriz Gonzalez-Castro, María Elena Medina-Mora, Humberto Nicolini

**Affiliations:** 1 División Académica de Ciencias de la Salud, Universidad Juárez Autónoma de Tabasco, Villahermosa, Tabasco Mexico; 2 Laboratorio de Genómica de Enfermedades Psiquiátricas y Neurodegenerativas, Instituto Nacional de Medicina Genómica, Mexico City, Mexico; 3 Hospital Psiquiátrico Infantil Juan N. Navarro, Servicios de Atención Psiquiátrica, Mexico City, Mexico; 4 Unidad de Encuestas y Análisis de Datos, Instituto Nacional de Psiquiatría Ramón de la Fuente Muñiz (INPRFM), Mexico City, Mexico; 5 Seminario de Estudios Sobre la Globalidad, Facultad de Medicina, Universidad Nacional Autónoma de Mexico (UNAM), Mexico City, Mexico; 6 División Multidisciplinaria de Comalcalco, Universidad Juárez Autónoma de Tabasco, Comalcalco, Tabasco Mexico; 7 Centro de Neurociencias Avanzadas, Departamento de Psiquiátrica del Hospital Psiquiátrico, Universitario Dr. José Eleuterio González, Universidad Autónoma de Nuevo León, Monterrey, Nuevo León Mexico; 8 Unidad de Cuidados Paliativos, Instituto Nacional de Cancerología, Mexico City, Mexico; 9 Laboratorio de Farmacogenética, Instituto Nacional de Psiquiatría Ramón de la Fuente Muñiz (INPRFM), Mexico City, Mexico; 10 División Multidisciplinaria de Jalpa de Méndez, Universidad Juárez Autónoma de Tabasco, Jalpa de Méndez, Tabasco Mexico; 11 Instituto Nacional de Medicina Genómica, Periférico Sur 4809, Arenal Tepepan, Tlalpan, 14610 Mexico City, Mexico

**Keywords:** Behavioural genetics, Genetic association study, Genomics, Genetics, Diseases, Neurology, Risk factors

## Abstract

The combination of substance use and psychiatric disorders is one of the most common comorbidities. The objective of this study was to perform a genome-wide association study of this comorbidity (*Com*), substance use alone (*Subs*), and psychiatric symptomatology alone (*Psych*) in the Mexican population. The study included 3914 individuals of Mexican descent. Genotyping was carried out using the PsychArray microarray and genome-wide correlations were calculated. Genome-wide associations were analyzed using multiple logistic models, polygenic risk scores (PRSs) were evaluated using multinomial models, and vertical pleiotropy was evaluated by generalized summary-data-based Mendelian randomization. Brain DNA methylation quantitative loci (brain meQTL) were also evaluated in the prefrontal cortex. Genome-wide correlation and vertical pleiotropy were found between all traits. No genome-wide association signals were found, but 64 single-nucleotide polymorphism (SNPs) reached nominal associations (*p* < 5.00e−05). The SNPs associated with each trait were independent, and the individuals with high PRSs had a higher prevalence of tobacco and alcohol use. In the multinomial models all of the PRSs (*Subs*-PRS, *Com*-PRS, and *Psych*-PRS) were associated with all of the traits. Brain meQTL of the *Subs*-associated SNPs had an effect on the genes enriched in insulin signaling pathway, and that of the *Psych*-associated SNPs had an effect on the Fc gamma receptor phagocytosis pathway.

## Introduction

Epidemiological studies have found high rates of substance use disorders in individuals with psychiatric disorders, and vice versa^1–6^. One characteristic of those presenting this comorbidity is an increase in the severity of the symptomatology: such patients usually present a diminished response to treatment, an exacerbation of psychotic, manic, and/or depressive symptoms, as well as an increased risk of suicide^7–10^, making them a highly vulnerable group^11^. The etiopathological mechanisms of this comorbidity have yet to be clarified, but there are some general hypotheses^12,13^. One of these is the diathesis-stress model, which holds that there must be a vulnerability (such as a genetic variability) in a stressful environment (such as a socioeconomic or family situation) for a comorbidity to appear^12^. With the help of technologies for analyzing genetic information, genome-wide association studies have identified a genetic predisposition to many psychiatric disorders, demonstrating a pleiotropic effect^14,15^. However, these genetic studies have focused on exploring associations with a single phenotype, without investigating the question of comorbidity^12^. There has been little study of genetic variants related to the appearance of comorbidity.

Recent genome-wide analyses of psychiatric disorders have also included little representation of Latin American populations^16^. The majority of studies are of Europeans, but Latin American populations, including that of Mexico, have a high degree of genetic mixing, and could thus be the source of new associations for some phenotypes^17–19^. A recent study in India, for example, found an association for schizophrenia that had not previously been reported in European populations^20^. Other studies have identified differences in non-Europeans from the polygenic risk scores (PRSs) derived from genetic associations based on European ancestry^16,21^. Because of the high degree of genetic mixing in Mexico, there is a need to find loci associated with the comorbidity. The objective of this study was thus to carry out an analysis of genome-wide association with the comorbidity of psychiatric symptomatology and substance use, substance use alone, and psychiatric symptomatology alone, in the Mexican population.

## Materials and methods

### Study population

This study included a total of 3914 individuals of Mexican descent, taken from two different samples: an epidemiological subsample based on population (*n* = 3393) from the Mexican Genomic Database for Addiction Research (MxGDAR), and a clinical subsample (*n* = 521) taken from the Mexican Genomic Database for Cross-Disorder Research (MeDaCrosR) (Table 1). The individuals were divided into four groups: (i) individuals with comorbidity (*Com*, psychiatric symptomatology and substance use), (ii) individuals with substance use alone (*Subs*), (iii) individuals with psychiatric symptomatology alone (*Psych*), and (iv) individuals without symptomatology or substance use (*Cont*).

**Table 1:** Overview of the Sociodemographic and Clinical Characteristics of the Samples.

	MxGDAR/Encodat^a^ (*n* = 3393)	MeDaCrosR^b^ (*n* = 521)	Total (*n* = 3 914)
**Gender**			
Female (*n*, %)	1495 (44.06)	214 (41.07)	1709 (43.66)
Male (*n*, %)	1898 (55.94)	307 (58.93)	2205 (56.34)
Age (years, mean, *sd*)	35.89 (15.42)	27.52 (15.85)	34.73 (15.74)
Comorbidity (*Com*, *n*, %)	423 (12.47)	181 (34.74)	604 (15.43)
Sustance use alone (*Subs*, *n*, %)	1165 (34.34)	NA	1165 (29.76)
Psychiatric symptomatology alone (*Psych*, *n*, %)	318 (9.37)	340 (65.26)	658 (16.81)
No symptomatology (*Cont*, *n*, %)	1487 (43.83)	NA	1487 (37.99)

^a^*MxGDAR *Mexican Genomic Database for Addiction Research. ^*b*^*MeDaCrosR *Mexican Genomic Database for Cross Disorder Research.

The population-based epidemiological subsample was derived from the Mexican Genomic Database for Addiction Research (MxGDAR/Encodat)^22^, which is based on a population recruited from the 2016 National Survey of Drug, Alcohol, and Tobacco Use^23^. In this subsample, individuals who had experienced at least one psychiatric symptomatology in their life (psychosis, hypo/mania, depression, anxiety, or obsessions/compulsions) and presented risky use of at least one psychoactive substance (tobacco, alcohol, or drugs) were included in the *Com* group (*n* = 423). The psychiatric symptomatology was evaluated using the screening section from the Diagnostic Interview for Psychosis and Affective Disorders (DIP-AD)^22,24^. Risky substance use was determined with the questionnaire from the 2016 Survey and consisted of meeting at least one of the following criteria: (A) lifetime smoking of at least 100 cigarettes; (B) excessive alcohol consumption (at least five drinks by men or four drinks by women on a single occasion in the month prior to the survey), possible abuse or dependence on alcohol in the previous year, or having stopped drinking because of problems with its use; or (C) consumption of illegal drugs or psychoactive medications without a doctor’s prescription at least six times, or consumption of at least two different illegal drugs or unprescribed medications. The *Psych*, *Subs*, and *Cont* groups were defined by their meeting one of the following criteria: *Psych* (*n* = 658) included individuals with psychiatric symptomatology but without risky substance use, *Subs* (*n* = 1165) included those without psychiatric symptomatology but with risky substance use, and *Cont* (*n* = 1487) included those with neither psychiatric symptomatology nor risky substance use.

The subsample from the Mexican Genomic Database for Cross-Disorder Research (MeDaCrosR) (*n* = 521) is a clinical sample of patients seen in different health centers in Mexico (Hospital Psiquiátrico Infantil “Juan N. Navarro,” Hospital General “Dr. Gustavo A. Rovirosa Pérez,” Hospital Universitario “Dr. José Eleuterio González,” and Hospital Psiquiátrico “Fray Bernardino Alvárez”). It includes patients with diagnoses of bipolar disorder, schizophrenia, and major depressive disorder. Patients were included in the *Com* group (*n* = 181) if they had been diagnosed with at least one psychiatric and one substance use disorder (abuse or dependence) and they were included in the *Psych* group (*n* = 340) if they had been diagnosed with a psychiatric disorder but not one of substance use. The criteria used were those of the DSM-IVR, and all of the diagnoses were made by a psychiatrist. The individuals were evaluated with different psychiatric scales, including the DIGS (Diagnostic Interview for Genetic Studies)^25^, the MINI (Mini International Neuropsychiatric Interview)^26^, and the Diagnostic Interview for Psychosis and Affective Disorder (DIPAD)^24^. Those evaluated with the MINI were also tested for tobacco dependence with the Fagestrom test^27^.

All participants provided written informed consent or assent, and the protocol complied with international norms and the Helsinki Declaration. The protocols for the MxGDAR/Encodat were reviewed and approved by the Research Ethics Committee of the Instituto Nacional de Psiquiatría (No. CEI/C/083/2015) and the Instituto Nacional de Medicina Genómica (No. 01/2017/I); the protocol for the MeDaCrosR was reviewed and approved by the Research Ethics Committee of the Instituto Nacional de Medicina Genómica (Nos. 23/2015/I and 06/2018/I).

### Microarray analysis

DNA was extracted by mouth swab or from blood cells using a modified salting-out method with the Puregen commercial kit (Quiagen, USA). The quality and integrity of the DNA was analyzed with a NanoDrop spectrophotometer (Thermofisher, USA) with 2% agarose gel. Genotyping was performed with the commercial microarray Infinium PsychArray (Illumina, USA). Fluorescence intensities were read with iScan (Illumina, USA) and converted to genotypes with GenomeStudio software (Illumina, USA). Genotyping of the MxGDAR and the MeDaCrosR was carried out in the high-technology microarray unit of the Instituto Nacional de Medicina Genómica, using the same protocol.

### Quality control of single nucleotide polymorphisms (SNPs)

Quality control was performed with Plink software^28^. SNPs were excluded if they had a variant calling greater than 95%, a minor allele frequency (MAF) greater than 5%, a Hardy–Weinberg equilibrium chi-square test *p*-value greater than 1e−5, and variants A/T or G/C (to avoid the flip strand effect). Individuals with a genotype call rate of less than 95% were excluded. To correct for cryptic relationships, all individual pairs with an identity-by-state value greater than 1.6 were marked, and the individual with the lowest genotype call rate was excluded^29^.

### Statistical analysis

#### Estimation of variance and correlation at the genome-wide level

The variance explained by the SNPs was calculated using a restricted maximum-likelihood analysis (GREML)^30,31^ with GCTA software^32^. Three models of explained variance were proposed, comparing: (1) *Com* with *Cont*, (2) *Psych* with *Cont*, and (3) *Subs* with *Cont*. A bivariate GREML analysis was also performed to evaluate genome-wide correlation between *Com* and *Subs*, *Com* and *Psych*, and *Subs* and *Psych*. All of the models were adjusted for age, sex, and ten principal components of global ancestry.

### Estimation of global ancestry

Estimation of global ancestry was performed with a principal components analysis using previously reported algorithms^33^ and the *PC-AiR* package^34^. The Human Genome Diversity Project (HGDP)^35^ was used as a reference for population-level genotypes. Only independent SNPs were included in this estimation; for this reason a process of linkage disequilibrium pruning (LD pruning) was carried out, using the following parameters: a window size of 50 kb, a step of 2, and a variance inflation factor of 5.

### Genome-wide genotype–phenotype associations

The genetic associations were carried out by means of multiple logistic regressions, adjusted for age, sex, and ten components of global ancestry as covariables. The logistic regressions considered (1) the *Com* group (MxGDAR, *n* = 423, MeDaCrosR, *n* = 181), a total of 604 cases; (2) the *Subs* group (MxGDAR = 1165); (3) the *Psych* group (MxGDAR = 318, MeDaCrosR = 340), a total of 658 cases; and (4) the *Cont* group (*n* = 3310). Three statistical contrasts were performed: (a) *Subs* and *Cont*, (b) *Psych* and *Cont*; and (c) *Com* and *Cont*. A *p*-value of 5.00e−05 was considered nominally associated and a value of 5.00e−08 was considered statistically significant on the genome-wide level. The logistic regressions were carried out in Plink^28^. After statistical contrasts, all of the variants were removed that had a MAF lower than 5.0% in cases or controls. An in silico functional annotation was also carried out of the associated SNPs using Variant Effect Predictor (VEP)^36^. In addition, the allelic frequencies of the associated variants were compared with the Genome Aggregation Database (GnomAD)^37^. A search in the GWAS atlas of the associated SNPs was performed for associations with brain-related phenotypes (psychiatric, substance use, and neurological disorders). Pathway analysis was carried out with the online ComPath tool^38^, using the KEGG (Kyoto Encyclopedia of Genes and Genomes)^39,40^ and Reactome databases. A clustering of paths was also performed^41^.

### Polygenic risk scores (PRSs)

Polygenic risk scores (PRSs) were calculated with SNPs associated with a significance threshold of *p* < 5.00e−05 for each statistical contrast, using Plink statistical software. Three PRSs were constructed based on the following statistical contrasts: (1) *Subs* and *Cont* (*Subs*-PRS), (2) *Psych* and *Cont* (*Psych*-PRS), and (3) *Com* and *Cont* (*Com*-PRS). The PRS is the sum of an individual’s genetic variation adjusted by the weight of every genetic variant associated with a specific *p*-value threshold^21^. Next, the standardized residual of the PRS was regressed on the ten principal components of global ancestry. Based on the distributions of these PRSs, the individuals were then divided into the following groups: (1) high PRS, where *Com*-PRS, *Subs*-PRS, or *Psych*-PRS was higher than the third quartile; (2) medium PRS, where any PRS fell between the first and third quartiles; and (3) low PRS, where any PRS was lower than the first quartile. The selected PRS was compared among the three groups (*High*, *Medium*, and *Low*) with an ANOVA. Next, the prevalence of psychiatric symptoms (psychosis, depression, anxiety, post-traumatic stress, obsession/compulsion, and hypo/mania) and substance use symptoms (ever used drugs, alcohol or tobacco use) was compared among the three groups with a chi-square test. These statistical comparisons were carried out with R^42^, and significance was established as *p* < 0.05. These analyses considered only the individuals from the MxGDAR, because these had a more in-depth phenotyping. Next, the accuracy of the PRSs to predict the phenotypes (*Com*, *Subs*, *Psych*, and *Cont*) was calculated with a multinomial regression using the *nnet* package. The sample was randomly divided into a training sample (70% of the individuals) and a test sample (30% of the individuals). A multinomial regression of the phenotypes in the training sample was performed on the three PRSs constructed, and the phenotype was then predicted in the test sample. A contrast table of the phenotype in the test sample and those predicted by the model were compared using the *caret* package, and the accuracy was calculated.

### Brain methylation quantitative loci (brain meQTL).

The possible functional impact of the associated SNPs on the brain was assessed by calculating brain methylation quantitative loci (brain meQTL), using a previously published database^43–45^ that included a total of 48 brain samples from individuals of Mexican descent. These were genotyped and DNA methylation levels were measured with microarrays. The genotyping was performed with the commercial Illumina PsychArray and the DNA methylation levels were determined using Illumina 450K BeadChips (Illumina, USA). A calculation of *cis*- and *trans*- meQTL was performed with KING software^46^, and *p* < 5.00e−8 was considered genome-wide statistically significant.

### Vertical pleiotropy

To explore whether the associated variants could perform vertical pleiotropy in the phenotypes with one exposure to an outcome, the following models were tested: (a) *Subs* exposure to the *Com* outcome, (b) *Psych* exposure to the *Com* outcome, (c) *Subs* exposure to the *Psych* outcome, (d) *Psych* exposure to the *Subs* outcome, (e) *Com* exposure to the *Subs* outcome, and (f) *Com* exposure to the *Psych* outcome. The vertical pleiotropy was tested using generalized summary-data-based Mendelian randomization (GSMR)^47^, with GCTA software. These analyses used SNPs in the GWAS at *p* < 5.0e−05 for each genome-wide contrast (*Subs* and *Cont*, *Psych* and *Cont*, and *Com* and *Cont*).

## Results

### Genome-wide explained variance and correlations

The genome-wide explained variance in the *Com* group was 17.31% (*V*_*g*_ = 0.1731, *se* = 0.0113, *p* = 1.16e−10), and in the *Psych* group it was 13.88% (*V*_*g*_ = 0.1387, *se* = 0.0154, *p* = 4.44e−16); in the *Subs* group it was not statistically significant (*V*_*g*_ = 0.00, *se* = 0.0113, *p* = 0.50). Genome-wide correlation, calculated by bivariate GREML, between *Com* and *Psych* was 0.4375 (*se* = 0.1884), between *Com* and *Subs* was 0.8324 (*se* = 0.0451), and between *Subs* and *Psych* was 0.0486 (*se* = 0.1542).

### Genome-wide associations

#### Genome-wide association in the *Subs* group

The genome-wide association analysis found no genome-wide statistically significant association between *Subs* and *Cont*; however, seven SNPs reached nominal association (*p* < 5.00e−05) (Table 2a, Supplementary Table S1). Of these, three were located on intergenic regions and four on intronic regions of the *LINC01622*, *LMO7*, *B3GNTL1*, and *C21orf91* genes. No enriched pathways were found. The only SNP reported in the GWAS atlas associated with a brain-associated phenotype was rs1304319, associated with being a morning person.

**Table 2:** Genetic loci associated with *Subs*, *Psych*, and *Com.*

SNP^a^	Band	Position	A1/A2^b^	MAF Subs^c^	MAF Cont	OR [95% CI] *p*-value	Gene
**(a) Subs**							
rs845885	6p25.3	6:1019362	G/T	0.3691	0.3186	1.36 [1.20–1.55] 1.82e−06	*LINC01622* (Intron)
rs2153893	6q23.3	6:137295402	T/C	0.2787	0.3264	0.76 [0.66–0.86] 4.55e−05	Intergenic
rs7048650	9p22.1	9:19397040	A/G	0.2894	0.2422	1.33 [1.16–1.52] 4.26e−05	
rs9530459	13q22.2	13:76250576	C/A	0.2174	0.1607	1.41 [1.20–1.66] 2.60e−05	*LMO7* (Intron)
rs9901757	17q25.3	17:80963331	C/T	0.4264	0.4684	0.77 [0.68–0.87] 1.97e−05	*B3GNTL1* (Intron)
rs1304319	18p11.32	18:1833682	T/C	0.2979	0.2436	1.33 [1.16–1.52] 4.92e−05	Intergenic
rs2824496	21q21.1	21:19171547	C/T	0.4569	0.5170	0.77 [0.69–0.88] 4.09e−05	*C21orf91* (Intron)
**(b) Psych**							
rs1098777	1q25.2	1:177508044	C/T	0.2122	0.2637	0.71 [0.60–0.84]4.75e−05	Intergenic
rs12140710		1:177613863	G/A	0.2836	0.3322	0.69 [0.59–0.81] 4.35e−06	
rs4652252		1:177625201	G/A	0.2353	0.2883	0.69 [0.59–0.82] 1.10e−05	
rs1923630		1:177649849	A/G	0.2583	0.3096	0.66 [0.57–0.79] 1.08e−06	
rs12139234		1:177669302	A/G	0.2635	0.3370	0.71 [0.61–0.83] 1.55e−05	*LINC01741* (Intron)
rs905488	2p14	2:68182025	T/C	0.3685	0.4501	0.72 [0.63–0.84] 1.07e−05	Intergenic
rs10208402	2q14.1	2:115203917	T/C	0.3247	0.2430	1.40 [1.19–1.64] 4.54e−05	*DPP10* (Intron)
rs7634577	3p25.1	3:13795677	T/C	0.5017	0.4369	1.34 [1.16–1.54] 4.67e−05	Intergenic
rs893883	3q23	3:142567327	A/G	0.4414	0.3592	1.37 [1.18–1.59] 3.97e−05	*PCOLCE2* (Intron)
rs6440116		3:142576455	T/C	0.4396	0.3592	1.38 [1.18–1.60] 3.14e−05	
rs13074870	3q26.32	3:177363666	C/T	0.4077	0.3303	1.36 [1.18—1.57] 2.88e−05	*LINC00578* (Intron)
rs6789946		3:177374031	C/T	0.3459	0.2803	1.36 [1.17–1.60] 3.97e−05	
rs2049156	4p15.1	4:29018173	A/G	0.2571	0.1976	1.43 [1.21–1.69] 3.30e−05	Intergenic
rs11097166	4q22.1	4:88611344	G/A	0.4866	0.4074	1.41 [1.22–1.62] 2.66e−06	
rs13132202	4q34.3	4:179125887	C/T	0.2052	0.2601	0.69 [0.58–0.82] 1.68e−05	
rs7701154	5q14.1	5:77716621	A/G	0.5128	0.4360	1.37 [1.18–1.60] 3.88e−05	*SCAMP1* (Intron)
rs3860112	5q31.1	5:134893439	C/T	0.4464	0.5088	0.73 [0.63–0.84] 1.47e−05	Intergenic
rs2317965	6p25.3	6:1545513	G/A	0.5461	0.4800	1.35 [1.17–1.55] 3.63e−05	*LOC102723944* (Intron)
rs7796255	7q34	7:138963364	A/C	0.2705	0.1940	1.46 [1.23–1.73] 1.19e−05	*UBN2* (Intron)
rs10885147	10q25.2- 10q26.13	10:113144540	T/G	0.2906	0.3288	0.71 [0.61–0.84] 3.69e−05	Intergenic
rs4269860		10:113180191	C/T	0.3250	0.3627	0.71 [0.61–0.84] 2.65e−05	
rs4590798		10:113194854	G/A	0.2831	0.3217	0.71 [0.61–0.84] 4.19e−05	*LOC105378485* (Intron)
rs11527950		10:113248666	C/T	0.2680	0.3100	0.70 [0.59–0.82] 1.81e−05	Intergenic
rs3107346		10:113261163	A/G	0.3406	0.3891	0.71 [0.61–0.83] 1.87e−05	
rs11195620		10:113287674	T/C	0.3792	0.4284	0.70 [0.60–0.82] 6.39e−06	
rs2138554		10:113310787	T/G	0.2953	0.3452	0.69 [0.59–0.81] 4.64e−06	
rs10885243		10:113445582	A/G	0.3107	0.3481	0.72 [0.62–0.84] 4.84e−05	*LOC105378486* (Intron)
rs1797		10:119705438	T/C	0.4264	0.3513	1.36 [1.18–1.58] 3.95e−05	Intergenic
rs10901874		10:126895917	T/C	0.2647	0.2017	1.42 [1.20–1.67] 3.74e−05	
rs11608012	11p15.1	11:21617059	A/G	0.3874	0.3088	1.42 [1.22–1.65] 3.74e−05	
rs1783235	11q23.3	11:116050271	G/A	0.2998	0.3612	0.72 [0.62–0.84] 3.25e−05	
rs1824603	13q13.3	13:35955344	G/A	0.2454	0.1834	1.45 [1.23–1.72] 1.37e−05	*NBEA* (Intron)
rs1964449	13q14.3	13:54034731	A/G	0.5226	0.4519	1.34 [1.17–1.55] 4.69e−05	Intergenic
rs589258	13q31.3	13:94807356	T/C	0.3585	0.2929	1.37 [1.18–1.59] 4.08e−05	*GPC6* (Intron)
rs608624	17q11.2	17:16404247	T/C	0.2956	0.2184	1.44 [1.22–1.70] 1.77e−05	Intergenic
rs8067381	17q12	17:36578907	T/C	0.1315	0.0797	1.64 [1.30–2.07] 3.44e−05	*ARHGAP23* (Intron)
rs2027670	18p11.23	18:7732141	G/T	0.3865	0.4691	0.70 [0.61–0.82] 3.52e−06	*PTPRM* (Intron)
rs658864		18:7734917	G/A	0.4231	0.3454	1.41 [1.22–1.64] 6.16e−06	
rs762416	21q22.3	21:45267134	T/C	0.3291	0.4075	0.71 [0.62–0.83] 6.11e−06	Intergenic
rs2070446	22q11.23	22:24035970	C/T	0.2605	0.2044	1.41 [1.20–1.67] 4.88e−05	*RGLP4* (p.His241Asp)
**(c) Com**							
rs12747494	1p22.2	1:91944137	C/T	0.2354	0.1910	1.56 [1.30–1.86] 1.40e−06	Intergenic
rs953855	2p12	2:77123866	G/T	0.4630	0.4912	0.71 [0.62–0.83] 1.29e−05	*LRRTM4* (Intron)
rs13033902		2:77264354	C/T	0.1952	0.2453	0.68 [0.57–0.82] 3.46e−05	
rs4848094		2:121400276	C/T	0.3853	0.3193	1.40 [1.20–1.63] 2.53e−05	Intergenic
rs1039201	3p25.3	3:11731029	T/C	0.4212	0.4705	0.73 [0.63–0.84] 3.50e−05	*VGLL4* (Intron)
rs971515	3q26.1	3:161153006	C/T	0.4863	0.4016	1.37 [1.18–1.58] 2.81e−05	*LINC02067* (Intron)
rs10076602	5q33.2	5:153902050	T/G	0.1096	0.1306	0.61 [0.48–0.77] 4.24e−05	Intergenic
rs12702917	7p21.3	7:9550478	A/G	0.4513	0.3606	1.38 [1.19–1.61] 3.67e−05	
rs887060	7p14.1	7:43057398	C/T	0.3010	0.3614	0.72 [0.62–0.83] 3.14e−05	
rs4470979	7p11.2	7:57320738	A/G	0.1852	0.2218	0.66 [0.55–0.80] 2.19e−05	
rs7078706	10q26.3	10:131525017	T/G	0.2847	0.2103	1.51 [1.27–1.78] 1.94e−06	*MGMT* (Intron)
rs7118149	11p14.3	11:24700545	A/G	0.4675	0.3776	1.41 [1.21–1.65] 8.86e−06	*LUZP2* (Intron)
rs10778569	12q23.3	12:108087904	G/A	0.2659	0.3315	0.68 [0.57–0.81] 4.92e−06	*PWP1* (Intron)
rs6491403	13q32.2	13:98866841	A/G	0.4700	0.4166	1.37 [1.18–1.58] 3.62e−05	*FARP1* (Intron)
rs4238213		13:98869342	T/G	0.5094	0.4436	1.42 [1.22–1.65] 3.65e−06	
rs11071657	15q22.2	15:62433962	A/G	0.5146	0.4240	1.36 [1.18–1.58] 4.47e−05	Intergenic
rs150857		20:62173817	T/C	0.1355	0.1851	0.31 [0.18–0.52] 1.41e−05	*SHPK* (p.Glu215Gln)

^a^Single-nucleotide variant, dbSNP code. ^b^A1 = effect allele/A2 = no effect allele. ^c^Minor allele frequency of A1 allele. ^d^Effect, in silico predicted effect of the variant.

#### Genome-wide association in the *Psych* group

The comparison between *Psych* and *Cont* found a total of 40 SNPs associated at a nominal statistical level (Table 2b, Supplementary Table S2). Of these, 26 were intergenic, 13 were intronic, and one was a missense polymorphism. Associated SNPs were found on five long non-coding genes (*LINC01741*, *LINC00578*, *LOC102723944*, *LOC105378485*, and *LOC105378486*), eight in protein-coding regions (*DPP10*, *PCOLCE2*, *SCAMP1*, *UBN2*, *NBEA*, *GPC6*, *ARHGAP23*, and *PTPRM*); the missense polymorphism was a change of histidine for aspartic acid in the 241 position of the RGLP4 (p.His241Asp). No enriched pathways were found grouping these genes. The following SNPs were reported in the GWAS atlas as associated at a nominal genome-wide significance (*p* < 5.00e−05) to psychiatric or substance use phenotypes in other populations: rs12139234, rs10208402, rs7634577, rs13122202, rs10885147, rs4269860, rs4590798, rs3107346, rs10885243, rs1964449, rs589258, and rs608624.

The SNPs rs12139234 and rs1964449 have been associated with schizophrenia/bipolar disorder and depression, respectively, while rs10885147, rs4590798, rs589258, and rs608624 have been associated with alcohol consumption on a typical day, starting age of smoking, and frequency of memory loss due to alcohol consumption in the previous year. The following SNPs have been associated with other brain-associated phenotypes: rs10208402 (chronotype), rs10885243 (insomnia and intelligence), rs13132202 (educational attainment), rs4269860 (right superior frontal diusivities), and rs7634577 (superior corona radiata radial diusivities).

#### Genome-wide association in the *Com* group

The comparison between *Com* and *Cont* found 17 SNPs associated at a nominally significant level (Table 2c, Supplementary Table S3). Of the 17 SNPs, seven were intergenic, nine were intronic, and one was a missense polymorphism. The nine intronic variants were located in the protein-coding genes *LRRTM4*, *VGLL4*, *MGMT*, *LUZP2*, *PWP1*, and *FARP1*, and in the *LINC02067* long non-coding gene. The missense polymorphism was a change of glutamine for glutamic acid in the 215 position of the SHPK (p.Glu215Gln). No enriched pathways were found grouping these genes.

The GWAS atlas search found five SNPs (rs12747494, rs953855, rs13033902, rs971515, and rs12702917) previously associated with psychiatric or substance use phenotypes in other populations. The SNPs rs12747494, rs971515, and rs12702917 were associated with past tobacco smoking, weekly alcohol consumption, and frequency of failure in the past year to fulfill normal expectations due to drinking, while rs953855 and rs13033902 were associated with ease of getting up in the morning and being a morning person.

### PRS analysis of the different groups

The polygenic risk score analysis constructed different scores for each statistical contrast (*Com* and *Cont*, *Subs* and *Cont*, *Psych* and *Cont*), at a significance level of *p* < 5.00e−05 (at this significance threshold there is no overlap of SNPs between PRSs). *Subs-*PRS explained 1.17% of the phenotypic variance (Pseudo-*R*^2^ = 0.0171, *se* = 0.0400, *p* = 9.35e−09), *Psych-*PRS explained 3.93% (Pseudo-*R*^2^ = 0.0393, *se* = 0.0508, *p* = 1.61e−13), and the *Com*-PRS explained 4.54% (Pseudo-*R*^2^ = 0.0454, *se* = 0.0422, *p* = 2.42e−15). In the multinomial model, there was a statistically significant association of the three PRSs with every group (Supplementary Table S1). The three PRSs had an accuracy of 40.69% (CI_95%_ 37.81–43.62%) for the prediction of *Com*, *Subs*, and *Psych*. Next, the individuals were grouped based on PRS, and the differences in psychiatric symptoms and substance use patterns in individuals with different PRSs were evaluated (Table 3); these analyses included only individuals from the MxGDAR.

**Table 3:** Psychiatric symptoms and substance use in the MxGDAR subsample with different PRSs.

	High PRS (*n* = 1950)	Medium PRS (*n* = 399)	Low PRS (*n* = 1044)	Statistic (*p*)
**Polygenic risk scores**				
Comorbidity (*Com*-PRS)	0.30 (1.07)	− 0.02 (0.37)	− 0.60 (0.73)	15.63 (4.26e−10)
Substance use (*Subs*-PRS)	0.31 (1.08)	− 0.01 (0.35)	− 0.59 (0.69)	11.58 (1.51e−07)
Psychiatric (*Psych*-PRS)	0.30 (1.05)	− 0.01 (0.35)	− 0.62 (0.72)	13.34 (1.19e−08)
**Psychiatric symptoms**				
Hypo(mania)	194 (9.95)	46 (11.53)	90 (8.62)	3.50 (0.1740)
Post-traumatic stress	144 (7.38)	28 (7.02)	56 (5.36)	4.38 (0.1118)
Depression	166 (8.51)	31 (7.77)	64 (6.13)	5.23 (0.0733)
Anxiety	131 (6.72)	16 (4.01)	48 (4.60)	7.53 (0.0232)
Obsession/compulsion	71 (3.64)	10 (2.51)	27 (2.60)	2.85 (0.2396)
Psychosis	37 (1.90)	4 (1.00)	12 (1.15)	3.19 (0.2028)
**Substance use**				
Alcohol^a^	765 (39.23)	151 (37.84)	332 (31.80)	15.94 (3.45e−04)
Tobacco^b^	489 (25.08)	92 (23.06)	198 (18.97)	13.73 (1.04e−03)
Cannabis	331 (16.97)	57 (14.29)	156 (14.94)	2.42 (0.2978)
Cocaine	138 (7.08)	24 (6.02)	74 (7.09)	0.40 (0.8174)
Tranquilizers	38 (1.95)	10 (2.51)	15 (1.44)	2.18 (0.3366)
Crack	35 (1.79)	8 (2.00)	20 (1.92)	0.17 (0.9186)
Inhalants	36 (1.85)	9 (2.26)	14 (1.34)	1.83 (0.4003)
Hallucinogens	23 (1.18)	4 (1.00)	12 (1.15)	0.06 (0.9722)
Amphetamines	14 (0.72)	4 (1.00)	8 (0.77)	0.43 (0.8062)
Heroin	8 (0.41)	3 (0.75)	3 (0.29)	1.63 (0.4434)
Opioids	4 (0.21)	0 (0.00)	5 (0.48)	3.12 (0.2100)
Sedatives	4 (0.21)	1 (0.25)	1 (0.09)	0.61 (0.7364)

^a^Excessive alcohol consumption, ^b^lifetime smoking of at least 100 cigarettes.

There were statistically significant differences between individuals with different PRSs in lifetime prevalence of anxiety, lifetime smoking of at least 100 cigarettes, and problematic use of alcohol (excessive alcohol consumption, possible abuse or dependence on alcohol in the previous year, or having stopped drinking because of problems with its use).

### Brain meQTL analysis of associated variants

The brain meQTLs associated with *Subs*, rs4787483, rs1304319, rs2824496, and rs9901757, had an effect on 269 CpG sites, 48 of which were annotated to the gene promoter (Table 4a). The greatest association was for rs2824496 affecting cg21278102, annotated to the promoter of *HSBP1* (Table 4a). The genes affected by the brain meQTL associated with *Subs* were enriched for the insulin signaling pathway (hsa04910, adjusted *p* = 0.0225) and the mTOR signaling pathway (hsa04150, adjusted p = 0.0269). Of the 40 SNPs associated with *Psych*, 19 (rs10208402, rs10885147, rs10885243, rs1098777, rs11527950, rs12140710, rs13074870, rs1923630, rs2049156, rs2317965, rs4269860, rs4590798, rs4652252, rs589258, rs608624, rs6789946, rs762416, rs7701154, and rs905488) had a *cis* or *trans* effect on 643 CpGs sites, of which 61 were annotated to the gene promoter, with the greatest effect for the rs13074870 associated with cg26017930, annotated to *SKI* (Table 4b). The brain meQTLs associated with *Psych* were enriched for 50 pathways, including Axon guidance (hsa04360, adjusted *p* < 1.00e−4) and Fc gamma R-mediated phagocytosis (hsa04666, adjusted *p* = 1.00e−04). Of the 17 SNPs associated with the *Com*, seven (rs12747494, rs1039201, rs4470979, rs7078706, rs7118149, rs953855, and rs971515) had an effect on 91 CpGs sites, of which 25 were associated with the gene promoter. In these associations the greatest effects were seen by the rs12747494 affecting cg25100604 and cg275519869, CpGs sites annotated to the promoters of *PCBP1* and *FAM133B*, respectively (Table 4c). There were no enriched pathways for the genes where the brain meQTL associated with *Com* had an effect.

**Table 4:** Brain methylation quantitative loci (meQTLs) of the associated SNPs.

SNP	SNP position	CpG	CpG position	CpG gene	Beta	*se*	*p*-value
**(a) Subs**							
rs4787483	16:29885447	cg17945962	2:67793216	*–*	1.8154	0.3932	3.80e−06
rs2824496	21:19171547	cg21278102	19:60482693	*HSBP1* (promoter)	− 1.2032	0.2537	2.10e−06
**(b) Psych**							
rs10208402	2:115203917	cg09191731	16:22825045	*HS3ST2*	1.3293	0.2874	3.73e−06
rs13074870	3:177363666	cg26017930	1:2232165	*SKI *(*Promoter*)	1.2075	0.2555	2.28e−06
		cg13906646	6:166892920	*RPS6KA2*	− 1.2005	0.2555	2.61e−06
		cg26559829	16:85206154		− 1.1834	0.2555	3.61e−06
		cg06852461	1:207975183	*C1orf132*	− 1.1825	0.2555	3.67e−06
		cg04144365	2:188483529		− 1.2275	0.2555	1.55e−06
		cg06709297	2:10302453	*C2orf48*	− 1.2226	0.2555	1.70e−06
		cg07046426	2:237623902		1.1832	0.2555	3.62e−06
		cg10464312	2:66672687	*MEIS1*	1.1709	0.2555	4.57e−06
		cg06055229	5:124077965	*ZNF608*	− 1.1714	0.2555	4.53e−06
		cg06445944	5:67586927	*PIK3R1*	− 1.1734	0.2555	4.36e−06
		cg14196170	6:32063594	*TNXB*	− 1.2095	0.2555	2.19e−06
		cg26577169	6:166401611	*LINC00602*	− 1.1663	0.2555	4.98e−06
		cg16107105	7:150646703	*KCNH2*	1.1754	0.2555	4.20e−06
		cg09759289	8:28223456	*FBXO16*	− 1.1725	0.2555	4.43e−06
		cg26280695	10:106072439	*ITPRIP*	1.1991	0.2555	2.68e−06
		cg03447547	14:94577038	*IFI27*	1.2104	0.2555	2.16e−06
		cg19733938	14:102510359	*DYNC1H1*	− 1.1678	0.2555	4.84e−06
		cg23909173	14:94451388		− 1.1927	0.2555	3.03e−06
		cg05333442	16:58533742	*NDRG4*	− 1.1678	0.2555	4.84e−06
		cg00080125	17:59490612	*C17orf82*	1.1733	0.2555	4.37e−06
		cg00591333	17:79109909	*AATK*	− 1.1854	0.2555	3.48e−06
		cg19919217	17:38248104	*THRA*	− 1.2131	0.2555	2.05e−06
		cg14771451	22:18508297	*MICAL3*	− 1.1858	0.2555	3.45e−06
rs7701154	5:77716621	cg18281939	5:77783894	*LHFPL2*	− 1.1750	0.2495	2.49e−06
**(c) Com**							
rs12747494	1:91944137	cg25100604	2:70314405	*PCBP1* (Promoter)	− 1.3127	0.2844	3.91e−06
		cg08057136	2:207506976	*–*	− 1.3785	0.2844	1.25e−06
		cg27519869	7:92219804	*FAM133B* (Promoter)	− 1.3326	0.2844	2.78e−06
rs953855	2:77123866	cg13294780	4:140005755	*ELF2*	− 1.1535	0.2494	3.74e−06
rs1039201	3:11731029	cg10087374	8:21960455	*FAM160B2*	1.1584	0.2500	3.61e−06

### Vertical pleiotropy

Vertical pleiotropy between phenotypes was assessed at *p* < 5.00e−05 for the associated SNPs. Vertical pleiotropy was found in both exposures: *Psych* to *Com* (*bxy* = 0.3004, *se* = 0.0675, *p* = 8.61e−06, *nSNPs* = 12) and *Subs* to *Com* (*bxy* = 0.4079, *se* = 0.1109, *p* = 2.35e−04, *nSNPs* = 7). Reverse vertical pleiotropy was also found in *Com* to *Psych* (*bxy* = 0.3065, *se* = 0.0803, *p* = 1.37e−04, *nSNPs* = 10) and *Com* to *Subs* (*bxy* = 0.3724, *se* = 0.0747, *p* = 6.11e−07, *nSNPs* = 10). There was vertical pleiotropy in *Subs* to *Psych* (*bxy* = 0.3224, *se* = 0.1056, *p* = 2.26e−03, *nSNPs* = 7), and reverse vertical pleiotropy in *Psych* to *Subs* (*bxy* = 0.2953, *se* = 0.0629, *p* = 2.69e−06, *nSNPs* = 12). Of the 64 SNPs associated with any phenotype at nominal significance, 29 (45.32%) showed vertical pleiotropy of any type.

## Discussion

The comorbidity between psychiatric symptomatology and substance use leads to a significant impairment in affected individuals, but the evaluation of the phenotype has been little explored in genome-wide studies. Ours is one of the first genome-wide association studies to explore variants associated with this comorbidity, along with substance use and psychiatric symptoms alone, in the Mexican population.

Our study found that the evaluation of different phenotypes (*Subs*, *Psych*, and *Com*) could identify different patterns of associated variants, but that these associated variants are highly correlated and could also have pleiotropy, the effect of a single variant on different phenotypes or traits^48^. Genome-wide association studies have found a high degree of correlation between psychiatric disorders^49^, possibly because many of the associated genes could have pleiotropic effects between different psychiatric phenotypes. In this study we found vertical pleiotropy and genome-wide correlation of all phenotypes, suggesting that the associated variants could have an effect in *Subs*, *Psych*, and *Com*. These have been reported recently in a genome-wide study of lifelong cannabis use, which found a genetic correlation of the phenotype with different substance use disorders as well as with other mental disorders, meaning that many of the associated genes could show a pleiotropic effect in these phenotypes^15^. Other studies have suggested that the categorization of individuals into discrete diagnoses may neglect the consideration of these individuals in terms of a broader phenotype, which may be occurring in comorbidity studies^50–54^. The use of genetic analysis could help us to better categorize individuals with comorbidity, as in our finding that individuals with higher polygenic risk scores had a higher prevalence of having smoked more than 100 cigarettes in their life and a higher prevalence of risky alcohol use. Interestingly, the variants found in the PRS analysis to be associated with *Com* and *Psych* have been associated in the GWAS atlas with alcohol and tobacco phenotypes. This may be the result of the larger sample size in our study of individuals using alcohol or tobacco. We thus believe that genome-wide association studies of comorbidity might include a greater diversity of substance use disorders in order to explore this phenotype-dependent difference in GWAS signals. If so, PRS results could be used in a clinical setting to screen for individuals with a greater risk of developing the comorbidity.

In order to assess the possible effects of the associated SNPs on the brain, we performed brain meQTL analysis of a previously published database of individuals of Mexican ancestry^43–45^. Some polymorphisms showed greater evidence of association with brain phenotypes, including rs10208402, rs12747494, and rs953855. The first, the intron SNP in the gene *DPP10*, for which we found an association with psychiatric disorders, has been associated with chronotype^55–57^. This variant exerted a *trans* effect on a CpG site on gene *HS3ST2*, also associated with chronotype^55^. We also found an association of rs953855, the SNP in the intron of *LRRTM4*, with the *Com*; it too has been associated with chronotype. A recent gene-level analysis found an association of the *LRRTM4* loci with lifetime cannabis use^15^. These results suggest that the comorbidity could be associated with chronotype. The rs953855 in this study was a brain meQTL, associated in *trans* to a CpG site in the *ELF2* gene, which has been suggested as a sensor for the elevation of extrasynaptic glutamate, modifying the growth of functional dendritic spines^58^. Glutamatergic signaling is a regulator in the reward regions of the brain that maintain the habits of psychoactive substance use^59^. The use of such substances alters this signaling; the mechanisms depend on the substance used, but the great majority promote an increase in glutamate at the synaptic level, which leads to an increase in the activation of neuronal receptors and a glutamate-dependent excitotoxicity, a mechanism dependent on Ca^2+^^60–64^. The increase in excitotoxicity could generate changes in neuroplasticity, leading to an increase in drug-seeking behaviors and in the memories associated with drugs^65^. The intergenic SNP, rs127474, for which we found an association with the comorbidity, is also associated with ever/never used tobacco^57^. This SNP had a *trans* effect on two CpG sites located in the promoters on *PCBP1* and *FAM133B*. *PCBP1* is part of the DISC1 interactome^66^, which is essential in the development of brain cells, and alterations in this area could lead to neurodegenerative disorders^66–68^. Peripheral levels of DISC1 have been proposed as a marker of nicotine exposure^69^, supporting the possibility of an association between rs127474 and tobacco-related behavior.

On the pathway/functional level, we found that brain meQTLs associated with *Subs* and *Psych* have a greater effect on brain pathways than those associated with *Com*. Those associated with *Subs* modified the insulin signaling pathway, while those associated with *Psych* modified the Fc gamma receptor mediated phagocytosis. Insulin signaling in the periphery plays a crucial role in the homeostasis of plasma glucose levels, but the effect of insulin on the central nervous system is less recognized. Insulin in the CNS is involved in cell survival, neurogenesis, receptor trafficking, and neurotransmitter release/reuptake^70–73^. The insulin pathway has been associated with substance addiction in animal and human studies, and in integrative bioinformatics analysis of different omic data^74^. The mechanism underlying the effect of insulin in substance use is not fully elucidated, but evidence points to a dysregulation of dopamine in brain reward circuits^75–79^. The Fc gamma receptor mediated phagocytosis pathway, for which we found brain meQTLs associated with psychiatric symptoms, was recently associated with schizophrenia and bipolar disorder through analysis of transcriptomes and co-localization of GWAS signals^80^. Zhao et al. have suggested that the alteration of the Fc gamma receptor pathway could affect lysosomal function, and found that individuals with lysosomal dysfunction had greater manifestations of psychiatric symptoms^81^, suggesting its possible importance in the manifestation of this symptomatology.

The genome-wide estimated variance in the *Com* group was greater than in those who presented only one disorder, suggesting that the risk from genetic variability could be greater in this group. Interestingly, the explained variance in the *Subs* group was close to zero. This lesser explained variance could mean that the effects of the common variants analyzed in the microarray are not sufficient to capture the genetic effect in this group, possibly because of an underestimation, possibly because of the effects of uncommon variants not explored in this study. An examination of these phenotypes with other sources of genetic variation is thus needed, and of the way in which this genetic risk could interact with environmental factors such as exposure to trauma and social tolerance of substance use in producing the comorbidity.

Although this study identified associations that could be the basis for future functional studies of the relationship between genetic variability and comorbidity, some limitations should be noted. The main one is the use of two samples, one a population sample and the other clinical, where the criteria for defining the phenotype could be a source of heterogeneity. However, the inclusion of both populations increases the sample size and facilitates the identification of associations. Even with these limitations, we believe that this study, with the sample size it offers for investigation of the genome-wide association, provides important information for the understanding of the comorbidity between psychiatric symptomatology and substance use in the Mexican population.

This study found genetic associations of SNPs that modulate brain DNA methylation levels in genes involved in the insulin signaling pathway and Fc gamma receptor phagocytosis with the comorbidity between psychiatric symptomatology and substance use in the Mexican population. These results suggest new paradigms for understanding how genetic variability regulates comorbidity.

## Supplementary Information

Supplementary Table S1.

Supplementary Table S2.

Supplementary Table S3.
